# Phylogenetic Relationships of Southern African West Nile Virus Isolates

**DOI:** 10.3201/eid0808.020027

**Published:** 2002-08

**Authors:** Felicity J. Burt, Antoinette A. Grobbelaar, Patricia A. Leman, Fiona S. Anthony, Georgina V.F. Gibson, Robert Swanepoel

**Affiliations:** * National Institute of Communicable Diseases, Sandringham, Johannesburg, South Africa

## Abstract

Phylogenetic relationships were examined for 29 southern African West Nile virus (formal name *West Nile virus* [WNV]) isolates from various sources in four countries from 1958 to 2001. In addition sequence data were retrieved from GenBank for another 23 WNV isolates and Kunjin and Japanese encephalitis viruses. All isolates belonged to two lineages. Lineage 1 isolates were from central and North Africa, Europe, Israel, and North America; lineage 2 isolates were from central and southern Africa and Madagascar. No strict correlation existed between grouping and source of virus isolate, pathogenicity, geographic distribution, or year of isolation. Some southern African isolates have been associated with encephalitis in a human, a horse, and a dog and with fatal hepatitis in a human and death of an ostrich chick.

West Nile virus (formal name *West Nile virus* [WNV]) is a mosquito-borne member of the *Flaviviridae* family (genus *Flavivirus*), which was originally isolated from the blood of a febrile patient in Uganda in 1937 (1). The virus is widely distributed in Africa, Asia, and Europe and was recently spread to the Western Hemisphere, where its presence was recognized in the northeastern United States in 1999 (2,3).

After the initial isolation of the virus, sporadic cases and outbreaks of febrile disease were recorded in humans in Africa, the Near East, and Asia; the largest outbreaks occurred in Israel in 1950–1954 and 1957 and in South Africa in 1974 (4,5). Meningoencephalitis was first observed in elderly patients in Israel in 1957 and subsequently observed as a complication in young children in India (6,7). In 1962, the isolation of WNV from a horse with encephalitis was reported in Egypt; from 1962 through 1966, meningoencephalitis occurred in both humans and horses in a series of outbreaks in southern France (8,9). In 1983, four cases of hepatitis, two fatal, were attributed to WNV infection in the Central African Republic (CAR), a report that has been largely overlooked (10). A marked increase in the frequency and severity of outbreaks of human disease during the 1990s followed, often involving horses; epidemics occurred in Algeria, Romania, Morocco, Tunisia, Italy, Russia, France, Israel, and the United States (11–18). Moreover, the recent outbreaks in Romania, Israel, and the United States were characterized by concurrent deaths in birds (19,20). The virus circulating in the United States was found to be most closely related genetically to a WNV isolate associated with goose deaths in Israel in 1998, suggesting that the virus was imported into America from the Near East, either in an infected bird, mosquito, human, or other animal. The exact mechanism of the introduction will probably remain unknown (18,19).

In southern Africa, WNV was found to be widely endemic in areas where the principal vector, *Culex univittatus*, and avian hosts of the virus are present. Human infections tended to be sporadic; large epidemics occurred when unusually high rainfall or hot weather favored breeding of the vectors (21–23). Outbreaks were associated with concurrent epizootics in birds, as evidenced by antibody studies, and 13 species of experimentally infected wild birds supported replication of the virus without becoming overtly sick or dying (24–26). The largest epidemic occurred in 1974 and involved tens of thousands of human cases over a 2,500-km^2^ area of the Karoo and Northern Cape Provinces (5,21). A mean antibody prevalence of 55% in humans was recorded in the affected region after the outbreak; levels of 80% to 85% were recorded in some locations. In one town, 1,700 people sought medical attention. Infections were most frequently subclinical or associated with mild febrile illness characterized by rash, myalgia, and arthralgia. No human deaths were recorded, and no excess bird deaths were observed, although an antibody prevalence of 53% was detected in wild birds after the outbreak (5,21). A smaller epidemic occurred from 1983 through 1984 in association with an outbreak of Sindbis virus (formal name: *Sindbis virus*) infection in the Witwatersrand-Pretoria region of South Africa, and again no deaths were recorded (23). Since then, the number of human WNV infections confirmed in South Africa, mainly on the basis of detection of antibody response, has remained fairly constant at approximately 5–15 cases per year. Only a proportion of suspected cases are subject to laboratory investigation. Despite the apparently low level of virus activity, however in recent years, a few isolations of WNV have been made from patients with severe disease, including a fatal case of hepatitis in 1989 and nonfatal encephalitis in 2001 (National Institute for Communicable Diseases [NICD], unpub. data).

The apparent increases in the frequency of neurologic infections, human case-fatality rates, and horse and bird deaths in the Northern Hemisphere raised the question of whether a recent emergence of WNV strains with increased pathogenicities occurred or whether the virulence of the virus had previously been underestimated. Investigations with hemagglutination-inhibition kinetics, titer ratios from cross-neutralization tests, reactivity to monoclonal antibodies, and cDNA/RNA heteroduplex restriction enzyme digest profiles confirmed that strain differences occur but did not establish any links between variants and pathogenicity (27–30). More recently, phylogenetic analyses based on different regions of the genome have shown that WNV isolates form two well-supported lineages (19,31,32). Lineage 1 includes viruses from Africa north of the equator, Europe, Asia, and North America; Kunjin virus (formal name: Kunjin virus [KUNV]) from Australia constitutes a subtype of this lineage. Lineage 2 consists solely of viruses from Africa and Madagascar. These findings support the emergence of increased virulence in lineage 1. Lineage 2 isolates are thought to be associated with endemic infection of low virulence in Africa (18,19).

The South African prototype WNV isolate, H 442, was obtained in 1958 from the blood of a person with mild febrile disease who had been bitten by mosquitoes while catching birds in mist nets for arbovirus studies (33). This isolate is the only one from South Africa to have been included in a phylogenetic study, and its characterization as a member of lineage 1 (19) seems to be inconsistent with findings for other African isolates from south of the equator. However, apart from recent isolation of WNV from severe cases of human disease in southern Africa, isolations of the virus were associated with a fatal infection in a dog, a horse, and an ostrich chick (34; unpub. data, NICD). Hence, we were prompted to undertake phylogenetic investigation of southern African WNV isolates.

## Methods and Materials

### Virus Isolates

Phylogenetic analysis was performed on partial nucleotide sequence data from 52 WNV isolates, which were sequenced for the study and included 29 from various sources in southern Africa from 1958 through 2001 (Table 1). Twenty-three WNV isolates and KUNV and Japanese encephalitis viruses (JEV) for which sequence data were retrieved from GenBank were also analyzed (Table 2). Southern African isolations made at NICD included 8 human, 15 mosquito, 1 bird, and 2 sentinel animal isolates. Three isolates obtained by the Onderstepoort Veterinary Institute, Pretoria, included the horse and ostrich isolates, which originated from the Regional Veterinary Laboratory, Stellenbosch, Western Cape Province, and a dog isolate, which originated from the Veterinary Investigation and Research Laboratory, Gaborone, Botswana, and was initially described as *Wesselsbron virus* (WESSV) but was later found to be WNV (34; BJH Barnard, pers. comm.). No isolates from the 1974 epidemic could be located for this study. The isolates were stored at -70°C as freeze-dried 10% mouse brain suspensions, and low-passage material was selected for sequencing (Table 1). With the prototype isolate, H 442, stocks of freeze-dried material were sequenced at various mouse passage levels (2–7), and passaged 2 material was passaged 10 times in mice and sequenced.

**Table 1:** Twenty-nine southern African West Nile virus isolates (sequences to be submitted to GenBank)

Isolate	Yr of isolation	Passage level	Source	Location	GenBank Accession no.
H 442	1958	m2	human	Ndumo, Kwa-Zulu Natal, RSA^a^	AF514918
H 912	1964	m4	human	Middelburg, Mpumulanga, RSA	AF514919
H 1127	1968	m4	human	Johannesburg, Gauteng, RSA	AF514920
SPU 101/89	1989	m5	human	Bloemfontein, Free State, RSA	AF514921
SPU 116/89	1989	m3	human	Pretoria, Gauteng, RSA	AF514922
SPU 167/89	1989	m7	human	Ovambo, Namibia	AF514923
SA 381/00	2000	m1	human	Naboomspruit, Northern Province, RSA	AF514944
SA 93/01	2001	m1	human	Johannesburg, Gauteng, RSA	AF514945
An 2842	1958	m3	long-billed crombec	Ndumo, Kwa-Zulu Natal, RSA	AF51424
An 15228	1968	m4	sentinel pigeon	Olifantsvlei, Gauteng, RSA	AF514925
An 20587	1972	m3	sentinel hamster	Mopeia, Mozambique	AF514926
An 24630	1977	m5	dog	Gabarone, Botswana	AF514927
94034039	1994	m4	ostrich	Prince Albert, Western Cape, RSA	AF514928
9604058	1996	m4	horse	Somerset West, Western Cape, RSA	AF514946
Ar 4064	1962	m2	*Culex theileri*	Olifantsvlei, Gauteng, RSA	AF514929
Ar 4821	1962	m3	*Cx. univittatus*	Olifantsvlei, Gauteng, RSA	AF514942
Ar 5254	1963	m4	*Cx. univittatus*	Welkom, Free State, RSA	AF514930
Ar 5995	1963	m3	*Cx. univittatus*	Olifantsvlei, Gauteng, RSA	AF514943
Ar 6127	1964	m3	*Cx. univittatus*	Olifantsvlei, Gauteng, RSA	AF514931
Ar 6129	1964	m4	*Cx. univittatus*	Olifantsvlei, Gauteng, RSA	AF514932
Ar 6618	1964	m3	*Cx. univittatus*	Olifantsvlei, Gauteng, RSA	AF514933
Ar 7941	1965	m3	*Cx. univittatus*	Olifantsvlei, Gauteng, RSA	AF514934
Ar 7943	1965	m2	*Cx. univittatus*	Olifantsvlei, Gauteng, RSA	AF514935
Ar 7944	1965	m3	*Cx. theileri*	Olifantsvlei, Gauteng, RSA	AF514936
Ar 8352	1966	m3	*Cx. univittatus*	Olifantsvlei, Gauteng, RSA	AF514937
Ar 10825	1969	m2	*Cx. univittatus*	Bethulie, Free State, RSA	AF514941
Ar 10864	1969	m2	*Cx. univittatus*	Bethulie, Free State, RSA	AF514938
Ar 10893	1969	m4	*Aedes caballus*	Bethulie, Free State, RSA	AF514939
Ar 20758	1984	m4	*Cx. univittatus*	Rondebult, Gauteng, RSA	AF514940

^a^RSA, Republic of South Africa.

**Table 2:** Twenty-three West Nile virus isolates, plus Kunjin and Japanese encephalitis viruses^a^

Isolate	Yr of isolation	Source	Location	GenBank accession no.
HEg 101	1951	Human	Egypt	AF001568
TL 443	1952	na	Israel	AF205881
G 16919	1955	na	India	AF205885
MP 22	1959	*Coquilletidia metallica*	Uganda	AF001562
PaH 651	1965	Human	France	AF001560
ArB 310	1967	*Culex* sp	CAR	AF001566
ALG-ArDjanet	1968	*Culex* sp	Algeria	AF001567
AnMg 798	1978	*Coracopsis vasa*	Madagascar	AF001559
AnD 27875	1979	*Galago senegalensis*	Senegal	AF001569
ArA 3212	1981	*Cx. guiarti*	Ivory Coast	AF001561
HB 83P55	1983	Human	CAR	AF001557
ArMg 956	1986	*Cx. quinquefasciatus*	Madagascar	AF001564
ArMg 978	1988	*Cx. univittatus*	Madagascar	AF001574
HB 6343	1989	Human	CAR	AF001558
ArD 78016	1990	*Aedes vexans*	Senegal	AF001556
ArD 93548	1993	*Cx. neavei*	Senegal	AF001570
097-50	1996	*Cx. pipiens*	Romania	AF205880
96-111	1996	na	Morocco	AF205884
Isr 98-Goo1	1998	goose	Israel	AF205882
PaAn981	1998	na	Italy	AF205883
KN 3829	1998	*Cx. univittatus*	Kenya	AF146082
NY-99, 382-99	1999	Chilean flamingo	USA	AF196835
ArNa1047	unknown	*Ae. albothorax*	Kenya	AF001571
Kunjin	na	na	Australia	AF001572
JE SA 14	1954	mosquitoes	China	U04522

^a^Nucleotide sequence data were obtained from GenBank for inclusion in the present phylogenetic analysis; na, not available; CAR, Central African Republic.

 The bird, mosquito, and sentinel animal isolates (Table 1) were obtained during epidemiologic studies (21); the human isolates (Tables 1 and 3) were obtained from clinical specimens submitted to the Arbovirus Unit or the Special Pathogens Unit at NICD for the investigation of suspected cases of arbovirus infection or for the exclusion of African viral hemorrhagic fevers. In all instances, WNV was isolated from human serum samples by mouse inoculation, except for patient 5, from whom the virus was isolated from a liver sample taken at autopsy.

**Table 3:** Southern African human patients from whom West Nile virus isolates were studied

Patient	Isolate designation	Yr	Sex/ Age	Syndrome	Outcome
1	H 442	1958	M/26	Fever, rash, myalgia, arthralgia	Survived
2	H 912	1964	M/Ad^a^	Fever, rash, myalgia, arthralgia	Survived
3	H 1127	1968	F/Ad	Fever, rash, myalgia, arthralgia	Survived
4	SPU 101/89	1989	M/33	Fever, coagulopathy, hemoglobinuria, renal failure	Survived
5	SPU 116/89	1989	M/27	Necrotic hepatitis	Died
6	SPU 167/89	1989	M/22	Fever, rash, myalgia, arthralgia	Survived
7	AR 381/00	2000	F/68	Fever, rash, myalgia, arthralgia	Survived
8	AR 93/01	2001	F/21	Fever, rash, myalgia, encephalitis	Survived

^a^Ad, adult.

### Reverse Transcriptase Polymerase Chain Reaction (RT-PCR) and Nucleotide Sequencing of Amplicons

Freeze-dried mouse brain suspensions were reconstituted in water, and viral RNA was extracted for the RT-PCR by using the QIAamp Viral RNA kit (Qiagen, Valencia, CA) according to the manufacturer’s instructions. A 255-bp region of the E glycoprotein gene (genome positions 1402–1656) was amplified with primers designated WN132 and WN240, as described by Berthet et al. (32). The RT-PCR reactions were performed with the TITAN One Tube RT-PCR kit (Roche Diagnostics, Germany) according to the manufacturer’s instructions. The nucleotide sequences of the amplicons were determined with BigDye Terminator Cycle Sequencing Ready Reaction kits with AmpliTaq DNA polymerase FS (Applied Biosystems, Warrington, Great Britain) according to the manufacturer’s instructions. Sequences were obtained for both strands of the DNA amplicons by using each primer, WN132 and WN240, for confirmation of the nucleotide sequence. Products were purified by using Centri-Sep spin columns (Princeton Separations Inc., Adelphia, New Jersey) and analyzed with a 377 GenAmp automatic sequencer (Applied Biosystems).

### Phylogenetic Analysis

Editing and alignment of the nucleotide sequence data were performed with DNASIS for Windows Version 2.5 (Hitachi Software Engineering America, Brisbane, CA). The phylogenetic analysis was performed on a 227-bp region of the amplicons with a neighbor-joining distance method (unordered “p” parameter model), with Phylogenetic Analysis with Parsimony (PAUP) software version 4.0b4a for Macintosh (35). Bootstrap confidence intervals were calculated by 500 heuristic search replicates.

## Results

### Clinical Features of WNV Infections

The human isolates (Tables 1 and 3) were obtained from clinical specimens submitted to the Arbovirus Unit for the investigation of suspected cases of arbovirus infection or undiagnosed fever, except for the three isolates obtained from specimens submitted in 1989 from patients 4, 5, and 6 (Table 3) for the exclusion of African viral hemorrhagic fevers; tests for Marburg disease, Ebola fever, Crimean-Congo hemorrhagic fever, Rift Valley fever, Lassa fever, and hantaviruses were negative.

Patients 1–3 and 6–7 (Table 3) had benign WNV infections with fever, rash, myalgia, and arthralgia; specimens from patient 6 were submitted for the exclusion of viral hemorrhagic fever only because he had an outdoor occupation in Namibia with potential exposure to ticks, and thus Crimean-Congo fever was considered a possibility. Patient 4 also had an outdoor occupation, in Free State Province of South Africa and a definite history of exposure to mosquito bites. During the second week of a febrile illness, he had coagulopathy with abnormal prothrombin index and partial thromboplastin time, hemoglobinuria, pancreatitis, and renal failure requiring dialysis. He made a prolonged but full recovery.

 Patient 5, who lived on the northern outskirts of Pretoria, had fever, nausea and vomiting, epigastric pain, elevated blood and urine amylase, elevated blood urea and creatinine values, and markedly elevated transaminases. He was admitted to the hospital on the second day of illness with low fever and tender epigastrium. Tests for hepatitis A, B, and C and HIV were negative, and the patient died on the fourth day of illness. No lesions of the pancreas were observed at autopsy, but a massive liver necrosis was found, and WNV was isolated from a liver sample.

Patient 8 lived near NICD, a WNV-endemic focus with artificial lakes and dams, reed beds, mosquitoes, and large bird colonies. The patient was admitted to the hospital with a 2-day history of headache, fever, nausea, and dizziness. She had marked light sensitivity and terminal meningism. Cerebrospinal fluid cultures and blood cultures were negative. She had severe arthralgia, and after discharge from the hospital, a rash developed. She made an uneventful recovery. Laboratory investigation of her illness, with consequent recognition of WNV infection, was probably influenced by the fact that she was a relative of a member of staff of NICD; other cases of WNV-induced encephalitis may have been missed.

The dog from which a WNV isolate was obtained in Botswana (Table 1) was missing for 4 days and found in extremis. The dog had severe diarrhea, became comatose, had convulsions, and died (34). The dog was initially thought to have rabies, and subsequently a flavivirus isolated from brain tissue was thought to be WESSV, but ultimately was shown to be WNV (34; BJH Barnard, pers. comm.). The horse isolate (Table 1) was obtained from the brain of a 6-month-old Thoroughbred foal from a farm in Somerset West District, which died after exhibiting signs of nervous disease. The ostrich chick came from the Prince Albert district in the Western Cape Province and was part of a major ostrich farming area where death in young birds at approximately 2 to 3 weeks of age has been a problem in recent years.

### Genetic Relationships

A phylogenetic tree was generated from sequence data of 52 WNV isolates from 19 countries, plus KUNV and JEV, by neighbor-joining distance analysis with node values generated by 500 bootstrap replications (Figure). The topology shows two distinct lineages. Lineage 1 includes 16 WNV isolates from 13 countries in Europe, Africa, the Near East, India, and the United States, and KUNV from Australia. The Indian isolate and Kunjin virus appear in lineage 1 as monophyletic sister clades. Excluding the Kunjin virus, the maximum nucleotide sequence divergence exhibited in lineage 1 (21.5%), was between the Indian isolate G 167919 and CAR isolate ArB 310. Otherwise, the divergences in lineage 1 ranged from a maximum of 10.6% between CAR isolate ArB 310 and Ivory Coast isolate ArA 3212, to a maximum homology of 99.6% between a Senegal isolate AnD 27875 and Algerian isolate Ar/Djanet.

**Figure:**
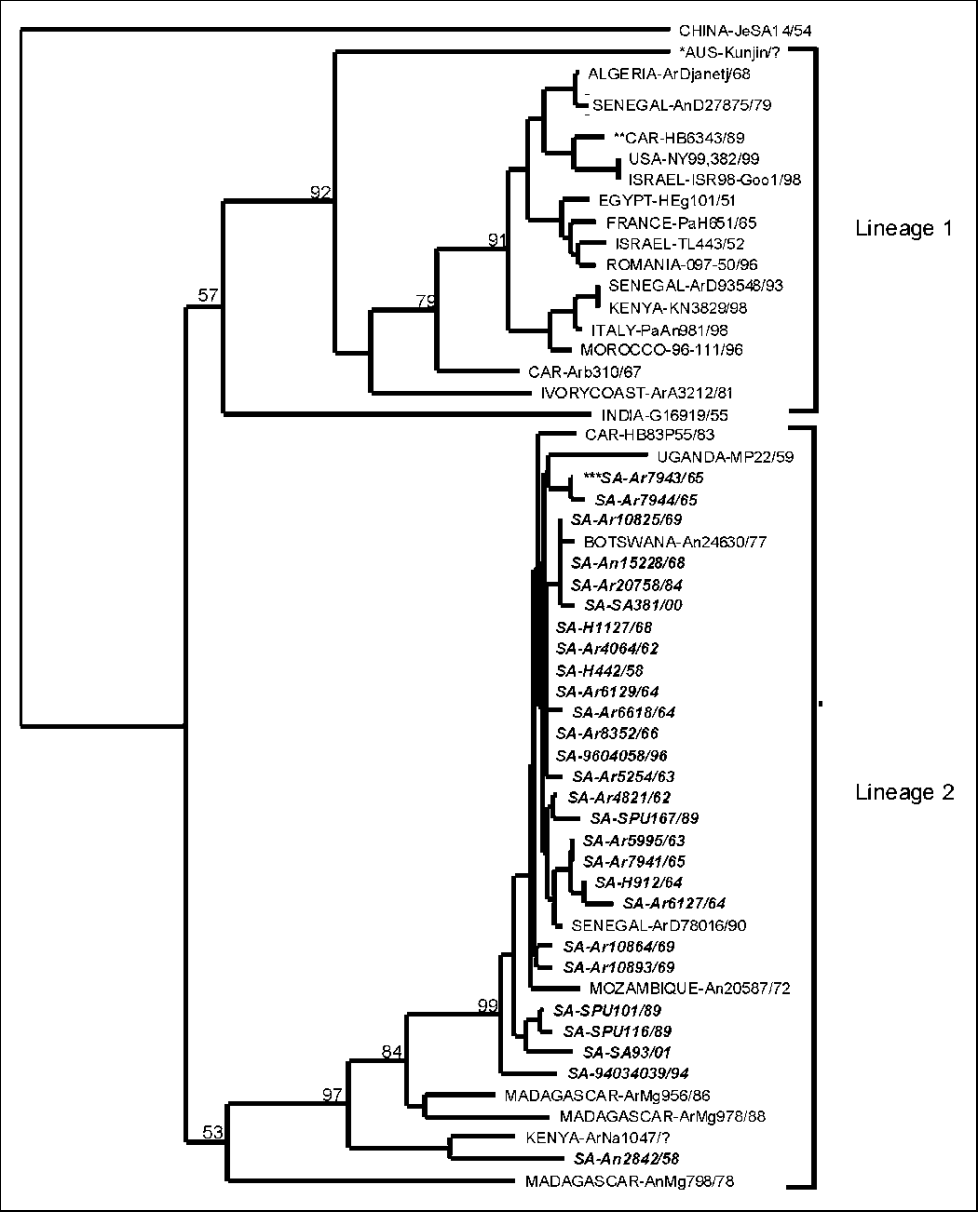
Genetic relatedness of geographically distinct West Nile isolates determined by using the nucleotide sequence data from a 227-bp region of the E gene. The tree was constructed with PAUP (35) by using neighbor-joining distance program. Node values were determined from 500 replicates. Isolates are labeled: country of isolation-strain identification/year of isolation. GenBank accession numbers are provided in Table 1 and 2. (*AUS, Australia; **CAR, Central African Republic; ***SA, South Africa). Sequences derived from West Nile virus isolates determined in the Special Pathogens Unit are shown in bold, italic type.

Lineage 2 includes 36 isolates from 7 countries in Africa, plus Madagascar. The maximum divergence of nucleotide sequences in lineage 2 (18.9%) was between a bird isolate from Madagascar AnMg 798 and several South African isolates from humans and mosquitoes. In the South African isolates, homology ranged from 86.3% to 100%. In some instances, identical isolates originated from different sources, years of isolation, or regions. The geographic dispersal of similar isolates was illustrated by the approximately 99.6% homology of Senagalese isolate ArD 78016 and several South African isolates. In contrast, geographic overlap of divergent isolates was also evident, as illustrated by the presence of strains from CAR, Senegal, and Kenya in both lineages.

## Discussion

The genetic relationships determined for isolates included in this study were consistent with previous publications in which isolates from different parts of the world fell into two lineages; one included a few African isolates and all the European, Asian, and North American isolates; the second lineage includes African and Madagascan isolates exclusively. In contrast to a previous finding (19), however, we determined that the South African prototype isolate, H 442, belonged to lineage 2 along with the other southern African isolates. Our findings were consistent in tests with material of different mouse passage levels, including material that had been stored at mouse passage level 2 at the time that the original isolation was made, supporting the conclusion that isolate H442 may have been misidentified in a laboratory abroad. Although no isolates from the 1974 South Africa epidemic could be located for inclusion in this study, they probably belong to lineage 2. All 29 southern African isolates tested belonged to lineage 2, even those that were isolated up to 16 years before or up to 19 years after the epidemic.

 The results of this phylogenetic study support previous conclusions that the close relationships between certain isolates from different countries and continents are compatible with local and long-range dispersal of the virus by migratory birds. On the other hand, the divergence between isolates of the two lineages indicates that different strains are circulating in some countries, such as CAR, Senegal, and Kenya. No genetic distinction appears between strains circulating in enzootic cycles and human outbreaks, and the wide range of suitable vector hosts probably facilitates the dispersal of WNV. The two extremes of bird migration routes, southern Africa and Eurasia, have divergent strains, whereas the central regions have a mixture of lineages 1 and 2. One possible explanation is that, because of the distances involved, the birds probably could not remain viremic from one extreme of their migratory route to the other. Ultimately viruses will likely pass from one region to the other.

Benign West Nile fever in humans is a febrile illness, with myalgia, arthralgia, lymphadenopathy, and often maculopapular or roseolar rash. Other documented signs and symptoms include nausea, vomiting, diarrhea, conjunctivitis, abdominal pain, pancreatitis, myocarditis, and hepatosplenomegaly. Few patients have serious complications such as acute aseptic meningitis, encephalitis, or necrotic hepatitis. Although many patients are reported to have severe illness with high case-fatality rates (4% to 13.3%) in recent epidemics in the Northern Hemisphere (16), serious disease occurs in 1% of infections (12,14,36,37). Moreover, most fatalities have been recorded in elderly or immunocompromised patients. The recent outbreaks have raised questions regarding strain variation and the possible emergence of enhanced pathogenicity. However, during a 1957 WNV outbreak in Israel, the death rate was 8.2% (4/ 49) in a group of elderly patients and 8.4% (35/417) in a epidemic in the same country in 2000 (16). Thus, the perceived virulence of the virus in recent epidemics may partly be because of the emergence or reemergence of existing strains of WNV in geographic locations with immunologically naive populations, high medical alertness, and active surveillance programs. When antigenic and molecular studies failed to demonstrate differences between WNV isolates from patients with hepatitis and benign disease in the CAR, rather than ascribing differences in clinical manifestation to virus strain variation, a new definition of disease spectrum to include liver involvement was considered (10,30).

The fact that no cases of severe disease were recognized in the large numbers of patients seen during the 1974 WNV epidemic in South Africa could indicate that isolates of lineage 2 lack virulence, but little clinical awareness of the pathogenic potential of arboviruses in general may also have played a role. The WNV epidemic coincided with the start of a major Rift Valley fever (RVF) epidemic in South Africa. Only in the next year (1975), when publicity surrounding the occurrence of Marburg disease in the country alerted clinicians to hemorrhagic disease, were specimens from hospitalized patients with unrecognized infections submitted for laboratory investigation (38). The invesigation proved for the first time that RVF could be a fatal disease in humans. Moreover, the recent isolations of WNV from patients with severe disease in recent years, including a fatal case of hepatitis and nonfatal mild meningoencephalitis during a quiescent period in virus activity, confirm that strains of lineage 2 can be pathogenic. Diagnosis of these cases owed more to the availability of appropriate laboratory services than to clinical recognition of WNV infection. Clearly, a need for greater awareness of the variety of symptoms, including hepatitis, associated with WNV throughout its distribution range is needed.

The occurrence of encephalitis in a dog in Botswana was followed by a serosurvey and pathogenicity trials in South Africa; the conclusion was that dogs are subject to WNV infection but probably do not play an important role in the epidemiology of the disease (39). The occurrence of WNV in horses in southern Africa is the subject of an ongoing investigation, but the recognition of an equine case of encephalitis remains an isolated event.

Although bird deaths characterized recent epidemics in the Northern Hemisphere, the disease appears to have spared African species in a New York zoo (19,20,40). Hooded crows had previously been shown to be susceptible to experimental infection with an Egyptian isolate (41), but experimentally infected adult wild birds of 13 species in South Africa had viremic infection without overt disease (24–26). The only death observed from an experimental infection in South Africa occurred in day-old domestic chicks; susceptibility declined with increasing age (PG Jupp, pers. comm.). WNV infection may have been responsible for the deaths observed in young ostrich chicks in the Western Cape Province, where the virus was isolated from a dead chick (Table 1), but many viruses, bacteria, intestinal parasites, and nutritional factors contribute to the death of young birds, and investigations are continuing. In conclusion, increased veterinary awareness of the pathogenic potential of the virus is needed.
